# Editorial: The neuroendocrine, autonomic and neuroinflammatory stress axes in cardiometabolic disease

**DOI:** 10.3389/fphys.2023.1293219

**Published:** 2023-09-25

**Authors:** Débora S. A. Colombari, Colin Sumners, Khalid Elsaafien

**Affiliations:** ^1^ Department of Physiology and Pathology, School of Dentistry, São Paulo State University, Araraquara, São Paulo, Brazil; ^2^ Department of Physiology and Aging, College of Medicine, University of Florida, Gainesville, FL, United States; ^3^ Centre for Integrative Cardiovascular and Metabolic Diseases, University of Florida, Gainesville, FL, United States; ^4^ Evelyn F. and William L. McKnight Brain Institute, University of Florida, Gainesville, FL, United States; ^5^ The Neuroscience Institute, Georgia State University, Atlanta, GA, United States; ^6^ Centre for Neuroinflammation and Cardiometabolic Diseases, Georgia State University, Atlanta, GA, United States

**Keywords:** neuroendocrine signaling, autonomic nervous system, neuroinflammation, cardiometabolic disease, stress

## 1 Introduction

**FIGURE 1:**
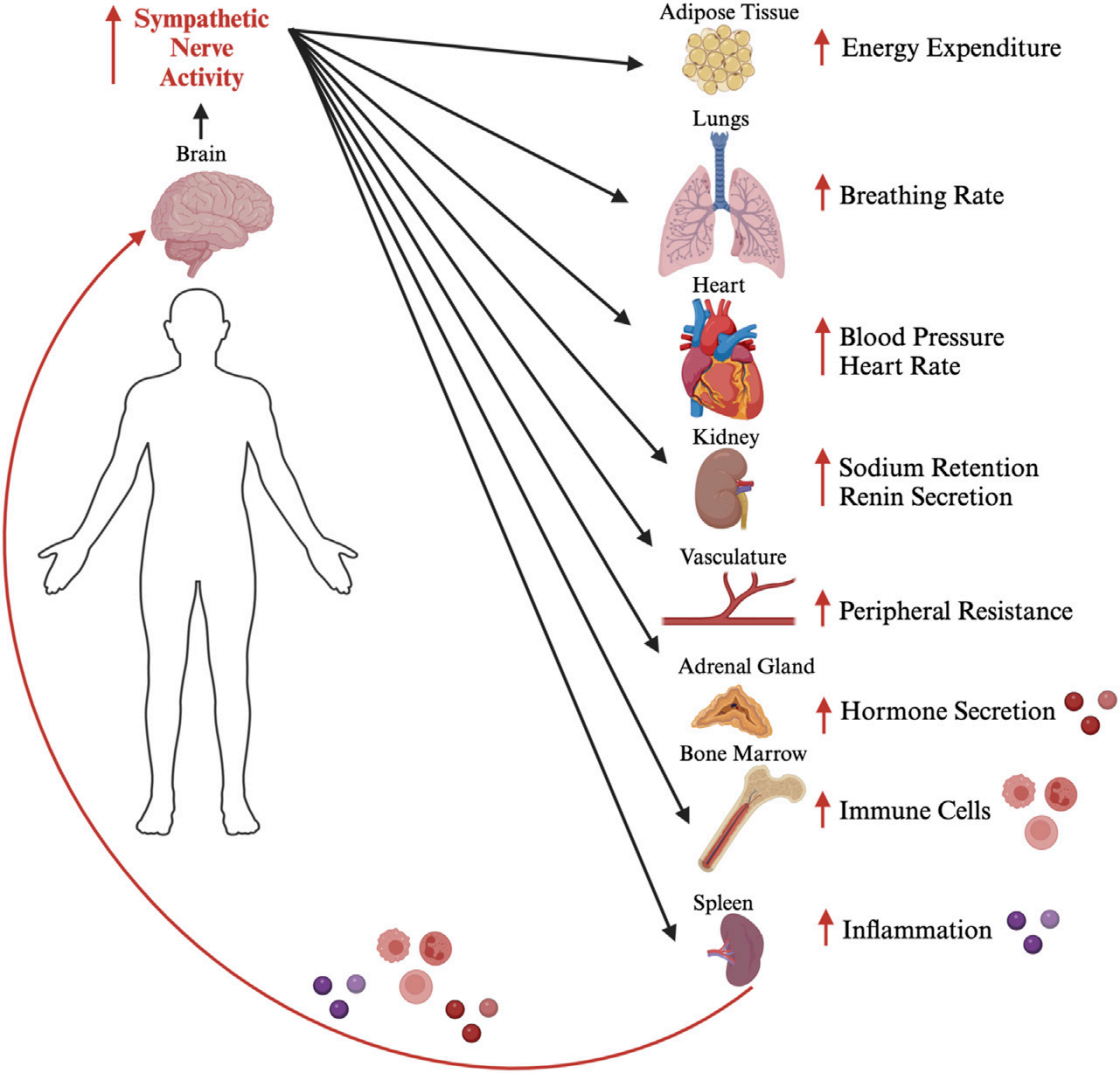
The neuroendocrine, autonomic and neuroinflammatory interplay. A schematic diagram depicting the role sympathetic nerves play in regulating different physiological outputs, which in turn can act on the brain in a feedback loop to modulate sympathetic nerve activity. This coordination of the different physiological functions through neuroendocrine, autonomic, and inflammatory mechanisms can become dysregulated resulting in a vicious positive feedback loop that leads to cardiometabolic disease. Figure generated by the authors using Biorender.com.

The survival of organisms requires homeostatic reflexes that stabilize the internal body environment in response to changes elicited by external and internal physiological stressors. The brain contains extensive neural networks spanning several specialized regions that control and regulate neuroendocrine, autonomic and neuroinflammatory output. These outputs dysfunction in cardiometabolic disease, and recent studies suggest that the interplay between all three outputs is integral in cardiometabolic physiology (Figure 1). The Paraventricular Nucleus of the Hypothalamus (PVN) contains neuroendocrine neurons that synthesize corticotrophin releasing hormone (CRH) to regulate the systemic release of corticosterone in response to stressors (Szafarczyk et al., 1986). The PVN also contains pre-autonomic neurons that influence sympathetic nerve activity to the cardiovascular organs to modulate blood pressure (BP) and heart rate (HR) (Strack et al., 1989). Furthermore, sympathetic nerve activity to the spleen influences the levels of circulating inflammatory cytokines (Katayama et al., 2022). Inflammatory cytokines can act on receptors expressed on neurons and glia within the PVN to regulate neuronal excitability and plasticity (Song et al., 2014).

The emergence of new technologies, such as CRISPR/Cas9 and Cre-LoxP recombination have enabled physiologists to selectively target neuronal populations of interest. A recent study investigated the neuroendocrine and autonomic populations of the PVN using such technologies (Elsaafien et al., 2021). It was demonstrated that the different neuronal populations of the PVN coordinate their activity to regulate cardiovascular function. CRH-synthesizing neurons signal inter-neuronally within the PVN to activate pre-autonomic neurons. This couples the increase in sympathetic nerve activity and systemic corticosterone secretion to BP elevations. The study demonstrated the importance of the interplay between the different neuronal populations to induce coordinated physiological responses.

This Research Topic provides an updated understanding of the interplay between neuroendocrine, autonomic and neuroinflammatory outputs in cardiometabolic disease. We have five excellent papers that include a brief research report, a mini review, a review, and two original research papers. These contributions span studies utilizing rodent models (*in vitro* and *in vivo*) and human studies. Of these articles, four were published in Frontiers in Physiology, while one was published in Frontiers in Neuroscience and have been viewed over 9,000 times so far. Physiologists are increasingly working towards providing a better understanding of neural circuits that regulate cardiometabolic function. This understanding is crucial for unraveling dysregulations that occur in cardiometabolic disease, allowing for the development of novel therapeutic targets.

## 2 The autonomic nervous system in cardiometabolic disease

In 1989, Arthur Loewy and others asked the question of what influences the releases of epinephrine and norepinephrine from the adrenal glands into systemic circulation (Strack et al., 1989). By applying pseudorabies virus into the left adrenal gland, they unraveled the sympathetic innervation of adrenal glands, which arises from five brain regions that include the PVN. Elia and Fossati provide an extensive review describing the autonomic innervation of the cardiovascular organs and their implication in cardiovascular disease. This understanding has led the authors to draw a link between autonomic dysregulation in cardiovascular disease and Alzheimer’s disease. It is well established that hypoperfusion of the brain leads to neuronal deterioration and accumulation of β-amyloid plaques that cause cognitive decline (Cermakova et al., 2015). The sustained adrenergic hyperactivity and robust sympathetic responses that lead to heart failure, result in reducing BP and causing hypoperfusion of the brain. This sustained adrenergic hyperactivity is accompanied by strong neuroendocrine stimulation that includes norepinephrine, renin, angiotensin, and aldosterone release (Leenen, 2007). Thus, the authors propose several interventions to the autonomic and neuroendocrine systems as therapeutic targets to mitigate Alzheimer’s risk in heart failure. Another link between cognitive function and autonomic regulation comes from Grosprêtre et al. where human participants performed motor imagery as a form of motor rehabilitation. The study demonstrated that motor imagery and posture can modulate spinal excitability, autonomic and cardiometabolic responses. Overall, these contributions demonstrate the importance of the interplay between cortical, autonomic, and neuroendocrine systems in cardiovascular disease.

## 3 The neuroendocrine system in cardiometabolic disease

Within the brain reside populations of neurons that influence the systemic release of endocrine signals. These hormones regulate physiological systems that are implicated in cardiometabolic disease. The study by Queathem et al. uses a novel transgenic mouse model to demonstrate the importance of the hormone estrogen in energy expenditure. They demonstrate a novel role of estrogen receptor β in white adipose tissue browning, via activation of protective adipocyte mitochondrial responses involving UCP1. This discovery highlights a novel pharmacological target for the treatment of metabolic disease. Similarly, the review by Pereira et al. proposes an important role that estrogen plays in sodium appetite. Excessive salt intake is well documented to contribute to hypertension. This review proposes that estrogen can act through the brain to alter salt palatability and inhibit sodium appetite, and proposes that neuroendocrine mechanisms that can be targeted to alleviate excessive sodium intake in hypertension.

## 4 Neuroinflammation in cardiometabolic disease

Several emerging studies have demonstrated an important role inflammatory cytokines and immune cells play in influencing the activity of neurons that regulate cardiometabolic function (Korim et al., 2018; Elsaafien et al., 2019; Elsaafien et al., 2020). These insights are implicated in cardiometabolic disease, where high grade inflammation is a hall-marker of the disease. The study by Oliveira et al. utilizes a transgenic mouse model to demonstrate that knock-in of angiotensin converting enzyme two in PVN CRH-synthesizing neurons rescues chronic hypoxia-induced pulmonary hypertension. This protection against pulmonary hypertension was mediated through autonomic modulation, counteracting vascular and lung inflammation, and reducing microglia activation in the PVN. The authors propose a vicious positive feedback cycle involving the neuroendocrine, autonomic and neuroinflammatory systems in pulmonary hypertension. This involves microglia activation that contributes to neuronal plasticity in CRH neurons of the PVN, leading to chronic sympathetic activation and immune cell recruitment which exacerbates this chronic pro-inflammatory and pro-sympathetic state in pulmonary hypertension.

## 5 Conclusion

This Research Topic of papers and reviews provides the reader with an overview of the interplay between neuroendocrine, autonomic and neuroinflammatory systems in cardiometabolic disease. We have highlighted some of the findings that help to unravel the complexity of this coordinated interplay. Investigating disease states requires an integrative approach, whereby the different systems at interplay are examined collectively. Such investigations are crucial for the development of novel effective therapeutics.
